# Using a checklist to facilitate management of long-term care needs after stroke: insights from focus groups and a feasibility study

**DOI:** 10.1186/s12875-018-0894-3

**Published:** 2019-01-04

**Authors:** Grace M. Turner, Ricky Mullis, Lisa Lim, Lizzie Kreit, Jonathan Mant

**Affiliations:** 1 Institute for Applied Health Research, University of Birmingham, Birmingham, Edgbaston B15 2TT UK; 2 Primary Care Unit, Department of Public Health and Primary Care, University of Cambridge, Strangeways Research Laboratory, Worts’ Causeway, Cambridge, CB1 8RN UK

**Keywords:** Stroke, Primary care, Long-term care, Rehabilitation, Quality of life

## Abstract

**Background:**

Long-term needs of stroke survivors are often not adequately addressed and many patients are dissatisfied with care post-discharge from hospital. Primary care could play an important role in identifying need in people with stroke.

**Aim:**

We aimed to explore, refine and test the feasibility and acceptability of a post-stroke checklist for stroke reviews in primary care.

**Design and setting:**

Focus groups (using a generic qualitative approach) and a single-centre feasibility study.

**Method:**

Five focus groups were conducted; three with healthcare providers and two with stroke survivors/carers. The focus groups discussed acceptability of a checklist approach and the content of an existing checklist. The checklist was then modified and piloted in one general practice surgery in the East of England.

**Results:**

The qualitative data found the concept of a checklist was considered valuable to standardise stroke reviews and prevent post-stroke problems being missed. Items were identified that were missing from the original checklist: return to work, fatigue, intimate relationships and social activities. Time constraints was the main concern from healthcare professionals and pre-completion of the checklist was suggested to address this.

Thirteen stroke survivors were recruited to the feasibility study. The modified checklist was found to be feasible and acceptable to patients and primary care clinicians and resulted in agreed action plans.

**Conclusion:**

The modified post-stroke checklist is a pragmatic and feasible approach to identify problems post-stroke and facilitate referral to appropriate support services. The checklist is a potentially valuable tool to structure stroke reviews using a patient-centred approach.

**Electronic supplementary material:**

The online version of this article (10.1186/s12875-018-0894-3) contains supplementary material, which is available to authorized users.

## Background

Stroke is the second leading cause of death and third leading cause of disability worldwide [1]. Globally, in 2013, there were approximately 25.7 million stroke survivors and 113 million disability-adjusted life years due to stroke [1]. As survival improves [2, 3], longer term care of stroke survivors is increasingly important. However, stroke survivors and their carers may feel abandoned and marginalised by healthcare services due to lack of proactive follow-up, unmet information needs, insufficient rehabilitation and lack of knowledge of support services [4, 5]. Many have unmet needs [6].

Primary care could play an important role in long-term care of stroke survivors, supporting access to community services and facilitating transfer back to specialist services if required. No formal primary care based model of care exists to support stroke survivors. A systematic and standardised approach to identify patients’ needs post-stroke and facilitate access to support services might improve patient experience.

An 11-item post-stroke checklist was developed by an international expert panel to standardise identification of long-term problems in a primary care setting and is endorsed by the World Stroke Organisation [7]. The checklist is feasible to administer and acceptable to patients and clinicians [8]. In the UK, health and social care needs of people after stroke and their carers should be reviewed in primary care at six months and then annually [9]. A post-stroke checklist used in primary care stroke reviews could promote active follow-up and reduce the marginalisation experienced by stroke survivors. However, primary care clinicians, stroke survivors and carers were not represented in the expert panel who developed the checklist. Our study aimed to: (i) explore views of healthcare professionals (generalists and specialists), stroke survivors and carers on the appropriateness and feasibility of a checklist in general and the content of the post-stroke checklist, and (ii) test the feasibility and acceptability of a modified version of the post-stroke checklist for primary care stroke reviews.

## Methods

This research forms part of a larger programme which aims to develop and evaluate a primary care based model to optimise stroke care post-discharge [10].

This paper reports focus groups and a feasibility study which were used to develop and test the feasibility a checklist to structure stroke reviews in primary care. The final checklist was informed through triangulation of these results with recommendations from a multidisciplinary intervention development group and patient and public involvement group.

### Focus groups

The aim was to explore the feasibility and acceptability of a checklist approach to facilitate management of long-term care needs post-stroke and the content of an 11-item post-stroke checklist [7]. A generic qualitative approach was used which aimed to generate a comprehensive summary of the key themes from focus group discussions through qualitative description methods [11, 12]. This is naturalistic approach that aims to understand and summarise the participants’ experience [13].

#### Participants

There were two participant groups: (i) healthcare providers, consisting of primary care professionals (general practitioners [GPs], practice nurses); community care professionals (allied health professionals); and volunteer sector representatives (e.g. Stroke Association workers), and (ii) stroke survivors and carers.

Stroke survivors were identified from general practices in the East of England in three socio-economic diverse areas: Cambridge, Bedford and Peterborough. Patients with confirmed stroke diagnosis, good understanding of English and no receptive aphasia were eligible. The study used a purposive sampling strategy to recruit a maximum variety sample to ensure a spread of age, socio-economic status, gender, disability and time from discharge.

GPs and practice nurses were identified through the Clinical Research Network-Eastern. Community care professionals, allied health professionals and volunteer sector representatives were identified through the research team’s community contacts.

#### Data collection and analysis

Five focus groups were conducted; three with healthcare providers and two with stroke survivors and carers. Two trained researchers facilitated the focus groups and a standardised topic guide was used. Focus groups were audio-recorded. Recordings were transcribed verbatim by a professional transcription service. Analysis was completed by a single researcher using a data-driven approach. The researcher familiarised themselves with the focus groups through reading the transcripts and listened to the audio recordings. Initial themes and reflections were noted. Thematic analysis was used to analyse the transcripts using inductive coding. Themes were established using an iterative process which included repeated reading, reviewing and refining themes and subthemes. The initial long list of themes and subthemes which emanated from the data were reduced through comparison across the different focus groups and participant within the groups, and focusing on the research aims [13].

### Feasibility study

The checklist was included in a six-month feasibility study which aimed to assess feasibility of a new model of primary care for stroke. The study was conducted at one general practice in Cambridgeshire and was non-randomised and non-controlled.

Stroke survivors were identified from the general practice stroke register. The eligibility criteria were: aged ≥18 years, stroke diagnosis, good understanding of English, capacity to give consent and not living in residential care.

#### Intervention

Participants attended a stroke review at their general practice, which was performed by a practice nurse who received training on use of the checklist. Prior to the stroke review, the checklist was sent to participants and they were asked to identify which items were relevant to them and choose three key needs, in order of importance. The participant’s prioritised needs were discussed in the review and an action plan agreed. The review also included a routine physical check-up.

#### Data collection and analysis

Two debriefing meetings were held with the practice nurse during the feasibility study and another at the end with the practice nurse, practice administrator and GP. Patients completed a feedback questionnaire immediately after the review and three months post-review. Quantitative data were summarised using descriptive statistics. Key themes were identified and summarised.

### Ethical approval

The focus groups were approved by the East of England Cambridge South Research Ethics Committee (REC) (REC Reference: 15/EE/0374). The feasibility study was approved by the West Midlands Edgbaston REC (REC Reference: 17/WM/0104).

## Results

### Focus group

Three focus groups were held with healthcare providers (ten specialists and nine generalists) and two were held with 12 stroke survivors and seven carers (Tables 1 and 2), between March and May 2016. On average the focus groups lasted 1 h 19 min (Appendix).

**Table 1:** Characteristics of stroke survivors and carers recruited to the focus groups

		Stroke survivors (*n* = 12)	Carers (n = 7)
Age (years)	Median [IQR]	67 [62, 78]	72 [70, 72]
	Range	49–81	65–77
Sex	Male	6	2
	Female	6	5
Deprivation status: IMD Decile	1–5 (most deprived)	1	1
	6–8	6	4
	9–10 (least deprived)	5	2
Time since stroke	< 1 year	4	N/A
	1–5 years	6	N/A
	> 5 years	2	N/A

*IMD* Index of Multiple Deprivation, *IQR* Interquartile Range

**Table 2:** Characteristics of healthcare providers recruited to the focus groups

		Healthcare providers (*n* = 19)
Age (years)	Median [IQR]	48 [43, 55]
	Range	28–58
Sex	Male	4
	Female	15
Time worked in stroke	< 1 year	0
	1–5 years	3
	> 5 years	16
Profession	General practitioner	5
	Occupational therapist	3
	Physiotherapist	4
	Practice nurse	4
	Speech and language therapist	2
	Assistant practitioner	1

IQR: Interquartile Range

#### Concept of the checklist

The concept was considered valuable by both participant groups (Table 3). Healthcare professionals thought it useful for structuring consultations and creating standardisation to address variability. The checklist was considered helpful to prevent problems being missed, particularly those that patients are reluctant to volunteer or that clinicians may not have considered. However, some healthcare professionals had negative perceptions of checklists as tick box exercises which inhibited patient-centred care. Stroke survivors and carers felt the checklist would promote proactive and stroke-specific follow-up. Primary care was perceived as reactive and patients/carers wanted appointments specifically related to stroke, but felt they had to “pester” primary care to get this.

**Table 3:** Themes from the focus groups

Concept of the checklist	Positive	“…and sometimes it’s something, you think “oh that, I may not have asked that if it wasn’t on a checklist”. So I do like checklists I have to say because it does remind you to ask things…” [Nurse] “…it just gives a structure to that consultation which I think means things are less likely to get missed really.” [Nurse] “… a checklist would be very useful because cut to the chase and gets to the way forward” [Stroke survivor]
	Negative	“And I’m slightly worried with a list that, you know, you sort of put it out nationwide and there’s going to be certain, doctors are going to be “another checklist, another”, you know, I can see that the first gut feeling that you’re going to have about it is a negative one which isn’t a good start.” [GP]
Content of the checklist	Missing items	Work: “And to me that was very, very important to try and get some normality back and for me normality was getting back to work.” [Stroke survivor] Carer needs: “Now I believe to solve the problem would be if when someone has a stroke there is a person who is nominated, possibly as a case worker probably, doesn’t matter, but someone who takes the relatives through what happens after someone has a stroke. Now when my wife was released from hospital after almost six months it was a case of “here’s a bag of tablets, bye” and it was great wheeling her out of the hospital. I got to the car, what do I do?” [Carer] Intimate relationships: “Intimate relationships as well because I think it’s not spoken about…” [Physiotherapist]
	Wording	“I suppose they could be, had been anxious and depressed for two years, doesn’t matter if they’re more anxious or depressed does it if they’re already, if that’s still not been managed that anxiety? Doesn’t matter if it’s more or less.” [Physiotherapist]
Barriers	Time constraints	“I liked the content but it’s long for, so if it was me seeing that patient in 10 min I would struggle to probably get through the first three questions in reality…” [GP]
	Inhibiting patient-centred care	“You know, so I think it’s not personalised and that’s why doctors won’t use a checklist like this, it will just seem too artificial. So there’s the time constraint but it just doesn’t work in terms of getting a patient’s confidence in an interaction, you know.” [GP]
	Resources	“I think that could cause certainly a resource issue for our team because we are absolutely tiny and our focus is, it tends to be the new strokes coming out of hospital, whereas we could end up seeing people a year down the line potentially because they do still have the problems and we know that.” [Occupational therapist]
	Raised expectations	“…a lot [of patients] will have some lasting permanent damage which they’ve probably been told several times that it’s unlikely to change or improve but the patient is wanting that. And here you’re asking has their, is their mobility still a problem and they’re saying yes and then you’ve put review to the community stroke team, that will generate a lot more referrals if you like to a rehab team or to physiotherapy or to occupational therapy but actually that person may not have the potential to improve. So you’re almost raising their expectations that something will happen or something will be done.” [Physiotherapist]

Many primary care clinicians already used some form of checklist/template; however, these were tailored for medical management and didn’t address the “holistic side” of stroke care. Checklists used by specialists did cover holistic aspects, but are too lengthy for primary care. Healthcare professionals emphasised the importance of short checklists and the need for a pathway to address problems identified. Both participant groups agreed that a checklist consultation needed to be completed by someone clinically qualified.

#### Content of the checklist

Items not in the checklist considered important include: return to work, confidence, changes in personality (such as anger problems), intimate relationships, driving, vision, swallowing, reading, skin integrity, benefits and support for carers. However, healthcare professionals acknowledged the checklist would be too long if all problems are included and some areas, such as benefits/ finances, are not within their area of expertise. They also acknowledge it may not be feasible to integrate carers’ problems and that these should be addressed separately.

Some healthcare professionals had concerns that the wording “since your stroke or last assessment…” may identify problems unrelated to stroke. Furthermore, phrasing the items to identify new or worsening problems, for example “do you feel more anxious or depressed”, was considered inappropriate because constant, ongoing problems may not be addressed.

#### Barriers

The main concern from healthcare professionals was length of time to complete the checklist and address all problems identified. To overcome this, they suggested the checklist could be pre-completed by patients and items could be prioritised.

In the context of multimorbidity, there were concerns about condition specific checklists creating additional workload. Other concerns were that the checklist may raise expectations for patients who will not benefit from further rehabilitation and that increasing referrals to support services may create resource problems.

#### Changes made to the checklist following the focus groups

A number of changes were made to the checklist, including changing wording, formatting and addition of new items (Table 4).

**Table 4:** Summary of development of the modified post-stroke checklist

	Philp’s et al 2013 post-stroke checklist	Initial modified post-stroke checklist	Final modified post-stroke checklist (Additional file 1)
Number of items	11	15	15
Items included	- Secondary prevention - Activities of daily living - Mobility - Spasticity - Pain - Incontinence - Communication - Mood - Cognition - Life after stroke - Relationship with caregiver	- Secondary prevention - Activities of daily living - Mobility - Stiffness - Pain - Incontinence - Communication - Mood - Cognition - Relationships with family - Fatigue - Intimate relationships - Work - Social activities - Other	- Secondary prevention - Activities of daily living - Mobility - Stiffness - Pain - Incontinence - Communication - Mood - Cognition - Relationships with family - Fatigue - Intimate relationships - Work - Social activities - Other
How items were selected	Delphi consensus methods with panel of stroke experts	Focus groups with stroke survivors, carers and healthcare providers (specialists and generalists)	Feasibility study
Administration	Administered by healthcare provider	Stroke survivor to complete prior to stroke review. Patient required to tick items relevant to them, prioritise 3 items and rank these in order of importance.	Stroke survivor to complete prior to stroke review. Patient required to tick items relevant to them
Changes made to the checklist		- Checklist is pre-completed by stroke survivors. - Wording adapted to be patient-friendly. - Items were worded as statements to identify prevalent/ new needs (rather than new/ worsening) - Two columns added for patients to (1) tick relevant items and (2) rank top 3 items. - 4 additional items added	- Requirement for stroke survivors to rank needs was removed

### Feasibility study

Thirteen stroke survivors were recruited between July and August 2017; 54% (7/13) were male, the mean age was 78 years (standard deviation [SD]: 8) and all participants were white. Reviews were conducted by one practice nurse and took place between six to 12 months post-stroke (23%; 3/13) or ≥ 12 months post-stroke (77%; 10/13).

#### Completion of the checklist

All 13 participants attended the review; however, four did not complete the checklist prior to the appointment. The main reason for not completing the checklist was participants feeling they did not have stroke related problems. Of nine participants that completed the checklist, two required help from family/friends. All nine participants completed the ‘needs’ column correctly and the number of items identified ranged from two to eight. However, none of the participants correctly followed the instructions on how to complete the second column which asked patients to rank their top three needs. For example, some participants did not complete this column and others put a value of 1–3 for all their needs identified in column one.

The mean number of items discussed during the review was three (range 1–5), the most common items were mobility (*n* = 7), fatigue (*n* = 5), social activities (*n* = 4) and secondary prevention (*n* = 4). Clear action points were agreed in 10 reviews. Referrals included: GP (n = 5), nurse (*n* = 3), physiotherapist (*n* = 2), falls assessment (*n* = 1), swallowing assessment (*n* = 1), diabetes prevention (*n* = 1) and exercise programme (*n* = 1).

Twelve participants completed the feedback questionnaire at baseline and 11 participants at three months post-review. Most participants rated the checklist as easy to complete and useful in both preparing for the review and during the review (Table 5). At baseline nine people were quite/very satisfied with the review, but this decreased to seven people at three months post-review.

**Table 5:** Participants feedback questionnaire

Baseline (*n* = 12)						
	N/A	very difficult	quite difficult	neither	quite easy	very easy
How easy was it to complete checklist?	0	0	0	2	5	5
	N/A	Not helpful at all	not very helpful	neither	quite helpful	very helpful
How useful was checklist in identifying needs?	1	0	1	2	4	4
How useful was checklist in preparing for review?	1	0	1	2	4	4
How useful was checklist during review?	1	0	1	4	6	0
	N/A	not at all satisfied	not satisfied	neither	quite satisfied	very satisfied
How satisfied were you with the review?	0	0	0	3	2	7
3 months post-review (*n* = 11)						
	N/A	much worse	somewhat worse	same	somewhat better	much better
Overall has care improved?	0	0	0	6	2	3
	N/A	Not helpful at all	not very helpful	neither	quite helpful	very helpful
How helpful was the review?	1	1	1	2	3	3
	N/A	not at all satisfied	not satisfied	neither	quite satisfied	very satisfied
How satisfied were you with the review?	1	0	1	2	3	4
	N/A	Strongly disagree	Disagree	Uncertain	Agree	Strongly agree
Would like review to become part of routine care?^a^	0	0	1	1	5	3

^a^1 missing response

*N/A* Not Applicable

#### Length of review

The mean review length was 44 min (SD: 9, range 25–55). Debriefing with the practice nurse identified the long review length was due to time management, rather than checklist completion, as the review was quite “chatty” and participants not pre-completing the checklist increased the review time. In addition, paperwork required for the research increased the appointment length and participants often asked questions about the research.

#### Changes made to the checklist following the feasibility study

Piloting suggested that asking patients to rank checklist items is too complicated; therefore, this was removed (Table 4). To save time, additional paperwork for the practice nurse was removed; we removed requirements to complete the checklist during the review if it had not been pre-completed; and nurse training was amended, including: (i) addressing time management issues; (ii) providing clearer instruction on using the checklist and recording outcomes; and (iii) discussing how to address patients asking about the research during the review.

## Discussion

### Summary

A patient-facing 15-item checklist, adapted from an 11-item post-stroke checklist [7], was developed to identify post-stroke problems and facilitate referral to appropriate support services. The revised checklist was developed using focus groups, a feasibility study and perspectives from key stakeholders. Initial piloting demonstrated that our modified checklist is feasible and acceptable to patients and primary care clinicians and resulted in agreed action plans.

### Strengths and limitations

Strengths of our study are that, through the focus groups and multidisciplinary intervention development group, perspectives from stroke survivors, carers, and healthcare professionals from primary, secondary and community care were included. Limitations are that the feasibility study was conducted at a single site, by one nurse and had a small sample size. The prevalence of unmet needs post-stroke differs between countries [8], so our checklist may not be generalizable internationally where differences in culture, healthcare systems and guidelines should be considered. Another limitation is that the project focused on stroke survivors needs in isolation and did not encompass the needs of carers and family members. Carers’ needs were brought up in the focus groups; however, it was suggested that it would not be feasible to integrate carers’ problems into the checklist. This is an important area for future research.

### Comparison with existing literature

Previous studies have demonstrated the feasibility and acceptability of the 11-item post-stroke checklist administered by primary care nurses and GPs in the UK [8], Italy [14] and Singapore [8]. Despite participants pre-completing our modified checklist in the feasibility study, our average appointment length (mean 44 min) was considerably longer that those observed in feasibility studies of the 11-item checklist: UK (mean 13 min, *n* = 42) [8], Singapore (mean 17 min, *n* = 100) [8] and Italy (73% < 5 min, *n* = 64) [14]. However, our feasibility data suggests the appointment length was largely due to participants’ interest in the research and additional paperwork for the practice nurse, rather than revisions to the checklist.

The 11-item post-stroke checklist is endorsed by the World Stroke Organisation, has been widely disseminated and is available in multiple different languages. However, primary care clinicians, stroke survivors and carers were not represented on the expert panel that decided the checklist’s content [7]. Our findings suggest that important problems from these perspectives were missing. Sexual dysfunction is relatively common post-stroke [15], but often unaddressed. Many healthcare providers do not raise sexual wellbeing with stroke survivors and do not see it as part of their role or feel it is inappropriate to raise [16]. Inclusion of sexual wellbeing in assessment tools provides an opportunity to raise the topic [16]. Fatigue is often overlooked despite many stroke survivors reporting this to be their main complaint [17]. Post-stroke fatigue has an impact on daily activities [18], return to work [19], rehabilitation [20] and mortality [21]. Return to work is important given that average age of first stroke has fallen over the past decade and more strokes are occurring in people of working age [22]. Stroke has long-term impact on social activities; six-years post-stroke, 65% of stroke survivors have lower levels of participation, compared to pre-stroke [23]. Social participation problems post-stroke are under-recognised by healthcare professionals [24]. Addition of an ‘other concerns’ section enables patients to report problems not otherwise included.

Other checklists have been developed in the UK to address long-term needs post-stroke. The 35-item Greater Manchester Stroke Assessment Tool (GM-SAT), includes guidance on action and was found to be feasible and acceptable for community stroke co-ordinators conducting six month reviews [25]. However, it is too time consuming for UK primary care (mean appointment length 74 min plus 33 min post-review for addressing needs). The Longer-term Unmet Needs after Stroke (LUNS) questionnaire is a 22-item self-completion questionnaire which is acceptable and reliable, but does not lead to action to address needs [26].

### Implications for research and practice

We have developed a patient-facing post-stroke checklist to structure stroke reviews, which provides a way to identify and address stroke survivors’ needs and ensures reviews are patient-focused. Our modified checklist includes four additional items, considered important to stroke survivors, carers and healthcare providers. Patients find it acceptable to complete prior to their appointment which has, in principle, the potential to reduce appointment length. Pre-consultation interventions, such a checklists, can increase question asking and patient satisfaction [27]. Thus, use of the checklist may increase active involvement of stroke survivors in their reviews and improve communication with healthcare professionals.

In our focus groups, healthcare providers expressed the importance of having a pathway to address stroke survivors’ needs once identified. There is a lack of information on availability and access to services for patients and carers which can lead to perceived marginalisation [4]. Therefore, when implementing a checklist for stroke reviews, training for primary care staff on stroke related long-term needs, appropriate action plans and knowledge of support services and referral pathways should be considered.

Our 15-item post-stroke checklist is currently being evaluated as part of a multifactorial primary care model for stroke in a cluster randomised controlled trial [28]. Although carers are included in the primary care stroke model, the American Heart Association/American Stroke Association recommends that specific interventions to target stroke caregivers are required [29, 30]. Further research to develop such initiatives is needed.

In conclusion, our 15-item post-stroke checklist, which involves patients pre-completing a checklist to identify their own needs, is feasible and acceptable to patients and primary care clinicians and results in agreed action plans. The checklist is a pragmatic approach to identify problems post-stroke and facilitate referral to appropriate support services and offers a way to structure stroke reviews using a patient-centred approach.

### Additional file

Additional file 1: Stroke Review Checklist. (DOCX 137 kb)

## Appendix

**Table 6:** Participants recruited to the focus groups

Participants			Length of focus group
Healthcare providers	Focus group 1	GP (n = 1) Physiotherapist (n = 2) Occupational therapist (*n* = 1) Practice nurse (n = 1) Assistant practitioner (n = 1)	1 h 30 min
	Focus group 2	GP (n = 3) Physiotherapist (n = 1) Occupational therapist (n = 1) Speech and language therapist (n = 1) Practice nurse (*n* = 2)	1 h 21 min
	Focus group 3	GP (n = 1) Occupational therapist (n = 1) Practice nurse (n = 1) Physiotherapist (n = 1) Speech and language therapist (n = 1)	1 h 16 min
Stroke survivors and carers	Focus group 1^a^	Stroke survivor (*n* = 8) Carer (n = 4)	1 h 23 min
	Focus group 2^b^	Stroke survivor (*n* = 4) Carer (*n* = 3)	1 h 3 min

^a^3 stroke survivor and carer couples, 5 single stroke survivors, 1 single carer; ^b^3 stroke survivor and carer couples, 1 single stroke survivor

## Abbreviations

GM-SAT: Greater Manchester Stroke Assessment Tool
GP: General practitioners
LUNS: Longer-term Unmet Needs after Stroke
REC: Research Ethics Committee
SD: Standard deviation
