# Supplementary material for: Using a checklist to facilitate management of long-term care needs after stroke: insights from focus groups and a feasibility study
Source: BMC Fam Pract. 2019 Jan 4;20:2. doi: 10.1186/s12875-018-0894-3 (PMC6318919; doi:10.1186/s12875-018-0894-3)
Supplement: Supplementary file 1 — Stroke Review Checklist. (DOCX 137 kb) [file 12875_2018_894_MOESM1_ESM.docx]

**Stroke Review Checklist**

This is a list of problems some people have after a stroke. This is to help you **think** about problems **you** may be having.

Under each heading there are some **examples**. This list does not cover everything. If you have a problem that is not listed here please write it under **number 15**.

**Please tick (*✓* )** **all the areas you have difficulty with**.

Tick here

**Please bring this completed checklist to your** **stroke review**.

| **1. Secondary Prevention** |  |
| --- | --- |
| I need advice on changes to lifestyle or medications for preventing another stroke. |  |
| **2. Activities of Daily Living (ADL)** |  |
| - I have difficulty dressing, washing and/or bathing. - I have difficulty preparing hot drinks and/or meals. - I have difficulty getting outside. |  |
| **3. Mobility** |  |
| - I am finding it difficult to walk. - I am finding it difficult to move safely around the house. |  |
| **4. Pain** |  |
| - I experience physical/muscular pain. - I have headaches. |  |
| **5. Stiffness** |  |
| I find that my arms, hands, and/or legs are stiff. |  |
| **6. Incontinence** |  |
| I am having a problem controlling my bladder or bowels. |  |

Tick here

| **7. Communication** |  |
| --- | --- |
| - I am finding it difficult to understand / communicate with others. - I have problems with speech, word finding or talking to others. - I lack confidence when talking to others either in person or over the phone. |  |
| **8. Mood** |  |
| - I feel anxious or depressed. - I feel that my personality since stroke has changed. |  |
| **9. Cognition** |  |
| I find it difficult to think, concentrate, or remember things. |  |
| **10. Relationships with Family** |  |
| My personal relationships with my family have become difficult or stressed. |  |
| **11. Fatigue** |  |
| - I feel tired most of the time or I get easily tired. - I find it difficult to concentrate and do things. |  |
| **12. Intimate relationships** |  |
| Since my stroke I have problems with sex. |  |
| **13. Work** |  |
| I am having problems at work or I would like support and advice on returning to work. |  |
| **14. Social activities** |  |
| I find it difficult to take part in hobbies or leisure activities. |  |

| **15. Have you noticed anything else that you are concerned about?** |
| --- |
|  |
